# Entropy-Driven Adaptive Decomposition and Linear-Complexity Score Attention: An AI-Powered Framework for Crude Oil Financial Market Forecasting

**DOI:** 10.3390/e28040392

**Published:** 2026-04-01

**Authors:** Jiale He, Chuanming Ma, Shouyi Wang, Yifan Zhai, Qi Tang

**Affiliations:** 1 School of Economics, Yunnan University, Kunming 650091, China; 20241040074@stu.ynu.edu.cn; 2 School of Law, Yunnan University, Kunming 650091, China; 20241030053@stu.ynu.edu.cn; 3 School of Business Administration and Tourism Management, Yunnan University, Kunming 650091, China; 4 Entrepreneurship Academy, Nanyang Technological University, Singapore 639798, Singapore; 5 School of Management and Economics, Tianjin University, Tianjin 300072, China

**Keywords:** financial market entropy, AI-driven crude oil price forecasting, variational mode decomposition, nature-inspired optimization, score attention mechanism, financial market uncertainty and risk assessment

## Abstract

The crude oil market has obvious financial entropy, and there are characteristics such as continuous uncertainty, multi-scale fluctuations and nonlinear state transitions. These characteristics bring challenges to the traditional prediction method. In this context, in order to improve the accuracy of energy financial market prediction, this study proposes an artificial intelligence-driven hybrid prediction framework, ALA-VMD-CASA. This framework is divided into three stages. First, with the goal of minimizing envelope entropy, ALA is introduced to adaptively optimize the hyperparameters of VMD, so as to generate informative sub-modes with reduced entropy. Next, the parallel prediction of each sub-mode is carried out by using the score attention mechanism based on the CNN autoencoder, and its linear time complexity can capture volatility clustering and sudden price fluctuations. Finally, the final price prediction is generated through the aggregation component. The empirical experiment of Brent crude oil spot prices from 2010 to 2025 shows that the ALA-VMD-CASA framework is superior to benchmark models such as ARIMA, RW, RWWD, LSTM, GRU, Transformer and Informer. Compared with the best standalone model, the proposed framework reduces the mean square error by more than 63% and obtains a perfect win rate in expanding-window evaluations. These results prove that the proposed framework is effective and robust for modeling financial entropy and improving energy price forecasting.

## 1. Introduction

Crude oil is an important global commodity and industrial foundation. Its price is a closely watched international market indicator, reflecting changes in the macroeconomy and the energy system [1,2]. Brent crude oil is the core of global pricing, reflecting changes in supply and demand and related to major benchmark prices [1,3]. However, the price fluctuations of Brent crude oil are not only caused by traditional supply and demand relations. Many factors contribute to price formation, such as geopolitical risks [4], policy or financial changes [5,6], energy, climate impacts [7], etc. They interact to generate complex market dynamics, making prices fluctuate and changing risks. Studies show that crude oil price series are non-stationary, have high-frequency fluctuations [8], and may also have nonlinear and chaotic characteristics [9].

Recent studies have also shown that entropy-based signal decomposition methods can enhance feature representation by explicitly minimizing entropy in the preprocessing stage, thus strengthening the link between information theory and financial forecasting methods [10,11,12,13,14,15]. In addition, for financial price series such as crude oil prices, the random-walk or no-change forecast provides a canonical benchmark that is often difficult to outperform in out-of-sample prediction tasks. Recent studies emphasize that the choice and correct specification of this benchmark are critical for interpreting forecasting performance [16,17].

Early studies on oil price forecasting relied on qualitative assessments and traditional econometric models, such as ARIMA, VAR, and GARCH [18,19,20]. These models remain useful for describing statistical regularities, volatility dynamics, and economic interrelationships in oil markets. For example, Cevik and Gillman (2025) used the local projection approach to examine the response of the oil market to macroeconomic policy shocks [21]. However, their value for economic interpretation should be distinguished from their predictive performance in out-of-sample forecasting settings.

Traditional econometric models typically depend on relatively strict parametric assumptions and linear statistical structures, which may become restrictive when oil markets experience structural breaks, parameter instability, or regime changes [22]. Recent studies further suggest that oil price formation exhibits pronounced nonlinear and time-varying characteristics, implying that more flexible models may be needed to capture its evolving dynamics [23]. At the same time, recent reassessments of oil price forecasting have shown that some previously reported forecasting gains are sensitive to benchmark specification and evaluation design, especially when the random-walk or no-change benchmark is omitted or inadequately defined [16,17,24]. This issue becomes even more relevant in high-dimensional environments characterized by structural change, policy adjustment, and regulatory shifts, where forecast robustness is critical [25]. Against this background, deep learning has introduced data-driven alternatives such as LSTM and GRU [26,27], which have been increasingly applied in energy price forecasting [28]. Nevertheless, when the original price series contains substantial noise and non-stationarity, directly feeding such sequences into deep neural networks may lead to unstable training and degraded forecasting performance.

To solve the problem, the decomposition-integration strategy has attracted attention in recent years [29,30]. In this framework, the original time series is first decomposed into components with different frequency characteristics, and then each component is modeled separately. This method helps the model capture the multi-scale dynamics of complex financial time series. Among the decomposition techniques, Variational Mode Decomposition (VMD) shows great potential in crude oil price prediction due to its ability to adaptively separate signals in the frequency domain [10,31].

Conventional trial-and-error procedures for parameter selection are inefficient and difficult to apply in large-scale forecasting environments. Consequently, meta-heuristic optimization algorithms have increasingly been introduced to determine optimal parameter configurations automatically [32]. Swarm intelligence methods such as Particle Swarm Optimization (PSO) and the Grey Wolf Optimizer (GWO) have been used to jointly optimize the parameters (K,α), reducing subjective bias in parameter selection. Nevertheless, these algorithms may still suffer from premature convergence and an imbalance between exploration and exploitation, which can hinder both global search capability and convergence efficiency [33,34,35].

At the subsequent forecasting stage, model design has gradually evolved from single LSTM or BiGRU structures toward more sophisticated hybrid frameworks incorporating attention mechanisms [36]. However, Transformer-based architectures, which rely on standard self-attention mechanisms, often encounter severe computational challenges in long-sequence time series forecasting tasks. Specifically, the computational complexity of self-attention grows quadratically with sequence length, i.e., $O(L^{2})$, which limits scalability when dealing with long historical sequences [37,38,39]. To alleviate these limitations, recent studies have explored lightweight combinations of convolutional neural networks (CNNs) and attention mechanisms, aiming to enhance local feature extraction while reducing computational overhead [40,41]. Within this context, the CNN autoencoder-based score attention (CASA) mechanism provides an efficient alternative. By leveraging lightweight CNN autoencoders to reconstruct latent representations of local temporal segments and assigning importance scores, CASA avoids global dot-product operations, reducing both time and memory complexity to linear order while improving inference efficiency [42].

Motivated by the above considerations, and aiming to jointly address the parameter sensitivity of VMD and the computational inefficiency of conventional attention mechanisms, this paper proposes a novel hybrid forecasting framework, termed ALA-VMD-CASA.

The main contributions of this study can be summarized in two aspects. First, we developed an adaptive optimization mechanism based on the ALA to determine the key parameters of VMD, thereby improving the stability and effectiveness of multi-scale signal decomposition while linking decomposition quality with an envelope entropy minimization objective. Second, we integrate the optimized VMD decomposition with a lightweight CASA to construct an efficient hybrid forecasting framework capable of capturing multi-scale temporal dependencies while maintaining linear computational complexity, which significantly enhances forecasting performance and robustness for crude oil price series.

The structure of the paper is as follows. Section 2 elaborates on the method, including VMD entropy-driven optimization based on ALA, the VMD framework, and the CASA mechanism. Section 3 describes the experimental design: covering data, models, evaluation indicators, results, ablation experiments, and robustness analysis (such as window rolling prediction, multi-step prediction, random initialization test). Section 4 conducts a summary, re-emphasizes the ability of the framework to reduce market entropy, and explores potential extension directions.

## 2. Methodology

This decomposition constructs the multi-scale structure in the data, and can analyze different time dynamics separately. An adaptive entropy-driven mechanism is set up to ensure that the decomposition can accurately reflect the underlying structure of the series and determine the settings for the decomposition. Based on the entropy-selected parameters, this method enhances the decomposition and characterization of complex and uncertain market dynamics. After decomposition, feature learning is further enhanced by the CASA mechanism based on the score-based attention structure. This model enhances the characterization of time patterns, improves the ability to capture dependencies from different time scales, and reduces the computational complexity. The obvious features after decomposition and the extracted feature representations are added to the prediction model, and then the prediction results of crude oil prices are generated.

### 2.1. Variational Mode Decomposition

VMD was proposed by Dragomiretskiy and Zosso [10]. It is an adaptive signal processing technique used to decompose signals into band-limited modes. For early decomposition methods such as EMD [43,44] and its ensemble empirical mode decomposition [45], VMD constructs the decomposition problem in a well-defined variable optimization framework. This formulation alleviates the problems of mode mixing and weak theoretical basis encountered by EMD-type methods. Unlike Empirical Mode Decomposition (EMD), VMD aims to decompose an input signal f(t) into *K* discrete sub-signals ${\left\{u_{k}\right\}}_{k=1}^{K}$, where each mode is compactly supported around its corresponding center frequency with a limited bandwidth.

To estimate the bandwidth of each mode, VMD constructs a one-sided frequency spectrum via the Hilbert transform and subsequently shifts the spectrum of each mode to the baseband. The signal decomposition procedure is therefore formulated as a constrained variational optimization problem, which seeks to minimize the sum of the bandwidths of all modes subject to the reconstruction constraint that the superposition of all modes equals the original signal. The resulting optimization problem can be written as follows:

(1) $$\min_{\left\{u_{k}\right\},\left\{\omega_{k}\right\}}\left\{\sum_{k=1}^{K}{∥\partial_{t}\left[\left(\delta \left(t\right)+\frac{j}{\pi t}\right)*u_{k}\left(t\right)\right]e^{-j\omega_{k}t}∥}_{2}^{2}\right\}\;s.t.\;\sum_{k=1}^{K}u_{k}=f,$$

In this formulation, $\left\{u_{k}\right\}$ denotes the set of decomposed modes, while $\left\{\omega_{k}\right\}$ corresponds to their associated center frequencies. The symbol δ(t) represents the Dirac delta function, and the operator * indicates convolution.

To address the constrained optimization problem described above, a quadratic penalty term α together with a Lagrange multiplier λ is introduced. This transformation converts Equation (1) into an augmented Lagrangian form without explicit constraints. The resulting saddle-point solution can then be efficiently obtained using the Alternating Direction Method of Multipliers (ADMM), which iteratively estimates the optimal set of variational modes.

From a functional perspective, the VMD process maps the input sequence to the set of decomposed components. For a discrete-time input sequence $x=\{x_{1},x_{2},\ldots ,x_{N}\}$, the resulting set of modes is largely determined by two parameters: the number of modes *K* and the penalty coefficient α. This relationship can be written as:

(2) $${\left\{u_{k}\right\}}_{k=1}^{K}=\mathrm{VMD}\left(x;\;K,\alpha \right),$$

which indicates that the characteristics of the resulting mode set are governed directly by the parameter pair (K,α).

**Figure 1:**
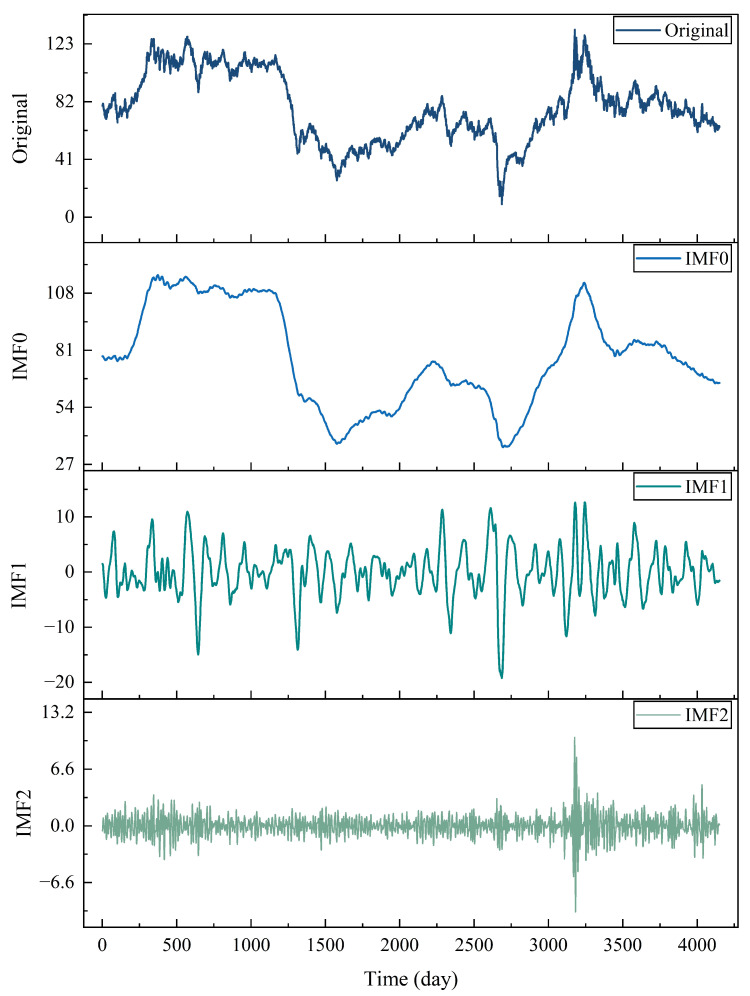
VMD results of the original time series. The original Brent crude oil price series is decomposed into several intrinsic mode functions (IMFs) with different frequency characteristics. IMF0 captures the low-frequency trend component, IMF1 represents medium-frequency oscillations, and IMF2 mainly reflects high-frequency fluctuations and noise.

Figure 1 presents the process of VMD decomposition. The original time series is decomposed into intrinsic mode functions (IMFs), and each IMF corresponds to a different frequency range. For example, IMF0 is mainly the low-frequency trend, IMF1 is the medium-frequency oscillation, and IMF2 is the high-frequency variable/noise. The result is that VMD adaptively separates each frequency band signal component in a non-recursive way.

From an economic perspective, the extracted IMFs can reflect the situation of crude oil price dynamics at different time scales. The low-frequency component IMF0 mainly reflects the long-term structural changes in oil prices. This change is related to factors driven by funds such as global supply and demand, changes in energy structure, macroeconomic cycles, inflation expectations, and U.S. dollar fluctuations. Over time, these factors change, shape the trend of oil prices, and also provide information for medium- and long-term forecasting.

### 2.2. Artificial Lemming Algorithm

Selecting appropriate values for the VMD parameters (K,α) is essential for obtaining reliable decomposition results. This task can be formulated as a nonlinear optimization problem in which parameter choices directly influence the quality of the extracted signal components. To address this issue in an automated manner, the present study employs the Artificial Lemming Algorithm (ALA), a recently proposed bio-inspired meta-heuristic introduced by Xiao et al. [46]. The algorithm is inspired by the collective foraging and migration behavior observed in lemming populations and provides an adaptive search strategy suitable for complex optimization problems.

The main mechanisms underlying ALA are outlined below.

(1) Energy control mechanism.

In the Artificial Lemming Algorithm, the behavioral dynamics of the population are regulated through an energy factor *E*. This factor gradually decreases as the iteration process proceeds and determines the transition between the global search stage and the local refinement stage of the algorithm. The energy level at iteration *t* is defined as:

(3) $$E=1-\frac{t}{T_{\max}},$$

where *t* denotes the current iteration number and $T_{\max}$ represents the maximum number of iterations allowed in the optimization process.

(2) Exploration phase (E>0.5).

When the energy level remains relatively high, the algorithm emphasizes exploration of the search space. In this stage, individual lemmings perform large-step movements in order to explore new regions and reduce the risk of being trapped in local optima. The position of the *i*-th lemming is updated according to

(4) $$x_{i}^{t+1}=x_{r}+C_{1}\cdot \left(x_{r}-x_{i}^{t}\right),$$

where $x_{r}$ represents a randomly selected position within the search space, and $C_{1}$ is a control parameter that regulates the magnitude of the movement.

(3) Exploitation phase (E≤0.5).

When the algorithm enters the exploitation stage, lemmings conduct intensive local searches in the vicinity of the currently identified best solution $x_{\mathrm{best}}$. The corresponding position update rule is given by

(5) $$x_{i}^{t+1}=x_{\mathrm{best}}+C_{2}\cdot \left(x_{\mathrm{best}}-x_{i}^{t}\right),$$

where $C_{2}$ denotes a convergence control parameter.

(4) Parameter mapping for VMD optimization.

Within the proposed framework, the position vector of each lemming directly corresponds to a candidate VMD parameter set. Specifically, let $x_{i}=\left(K_{i},\alpha_{i}\right)$. Through iterative updates of $x_{i}$, ALA minimizes a predefined fitness function, thereby identifying the globally optimal parameter combination $\left(K^{*},\alpha^{*}\right)$.

Through the above mechanisms, ALA is capable of efficiently converging toward the global optimum in a complex parameter space.

The complete ALA-based VMD optimization procedure is summarized in Algorithm 1.
**Algorithm 1** ALA-based Adaptive Optimization of VMD Parameters1:**Input:** Original time series x, population size $N_{p}$, maximum iteration number $T_{\max}$, search bounds of VMD parameters $K\in [K_{\min},K_{\max}]$ and $\alpha \in [\alpha_{\min},\alpha_{\max}]$.2:**Output:** Optimal VMD parameter set $\left(K^{*},\alpha^{*}\right)$.3:Randomly initialize the lemming population ${\left\{x_{i}\right\}}_{i=1}^{N_{p}}$4:For each lemming individual $x_{i}$, perform VMD on x and evaluate the fitness value based on envelope entropy5:Determine the current global best solution $x_{\mathrm{best}}$6:Set iteration counter t=07:**while **$t<T_{\max}\;$**do**8:   Compute the energy factor *E* according to Equation (3)9:   **for** each lemming individual $x_{i}$ **do**10:     **if** E>0.5 **then**11:        **(Exploration phase)**12:        Randomly select a position $x_{r}$ from the current swarm13:        Update $x_{i}$ using the exploration rule (Equation (4))14:     **else**15:        **(Exploitation phase)**16:        Update $x_{i}$ using the exploitation rule (Equation (5))17:     **end if**18:     Apply boundary constraints to ensure $K\in [K_{\min},K_{\max}]$, $\alpha \in [\alpha_{\min},\alpha_{\max}]$, and enforce $K_{i}$ as an integer19:   **end for**20:   Recalculate fitness values and update $x_{\mathrm{best}}$21:   Set t=t+122:**end while**23:**Return** the optimal VMD parameter set $\left(K^{*},\alpha^{*}\right)$

### 2.3. CNN Autoencoder Score Attention

Although Variational Mode Decomposition (VMD) alleviates the non-stationarity of the original time series, effective forecasting of each decomposed mode $u_{k}$ still requires accurate modeling of local morphological patterns and latent global dependencies. To address this issue, this study introduces the CASA mechanism. Originally proposed by Lee et al. in 2025, CASA replaces the quadratic-complexity dot-product attention in standard Transformers with a CNN-based linear-complexity attention mechanism, thereby significantly reducing computational overhead while preserving representational capacity [42].

**Figure 2:**
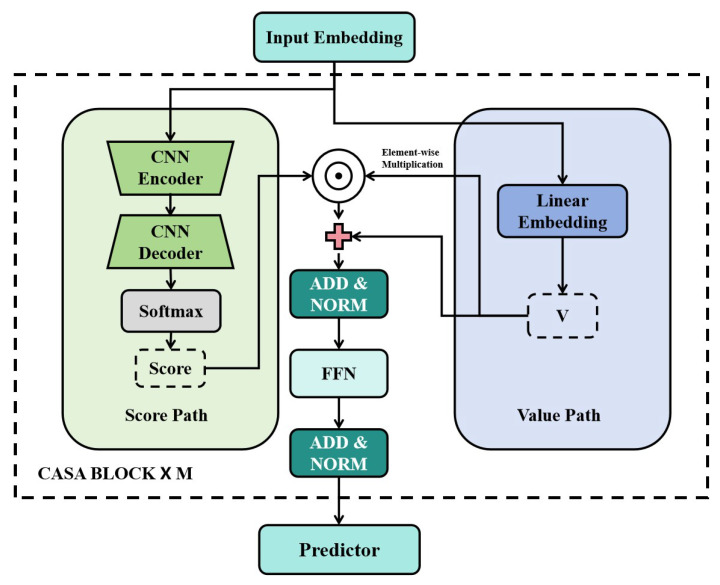
Architecture of the CASA module. The module consists of a score generation path based on a CNN encoder–decoder structure and a value generation path based on linear embedding. The attention output is obtained via element-wise multiplication of the score and value representations.

The CASA module consists of two parallel processing paths for the input embedding sequence *X*. The overall architecture of the CASA block is illustrated in Figure 2.

(1) Score generation path.

First, CASA constructs an attention score generation path using a one-dimensional convolutional neural network (1D-CNN) autoencoder to extract local morphological features from the input sequence. The encoder compresses local contextual information, while the decoder reconstructs the latent feature representation. The reconstructed output is then normalized by a Softmax function to produce the attention score vector s, given by:

(6) $$s=\mathrm{Softmax}\left(\mathrm{Decoder}(\mathrm{Encoder}(X))\right).$$

(2) Value generation path.

In the value generation path, the input sequence *X* is projected into a value representation *V* through a linear embedding layer:

(7) $$V=W_{V}\cdot X.$$

(3) Feature fusion stage.

At the feature fusion stage, the final output *O* is obtained via element-wise multiplication, whereby the attention scores derived from local morphological features are used to reweight the value representation:

(8) O=s⊙V,

where “⊙” denotes the Hadamard (element-wise) product. The resulting output *O* is subsequently fed into a feed-forward network (FFN) to generate the final forecasting results. The CASA block adopts a Transformer-style residual structure with Add and Norm operations to enhance training stability and representation capacity.

As a result, CASA introduces a more appropriate inductive bias for modeling complex and non-stationary energy price series, complementing the multi-scale decomposition achieved by VMD and further improving the overall predictive stability of the proposed framework.

### 2.4. The Proposed AI-Driven Entropy-Aware Forecasting Framework

This section presents the overall architecture of the proposed ALA-VMD-CASA ensemble forecasting framework. The framework establishes an end-to-end mapping from parameter optimization to final forecasting through four sequential stages. The logical connections among modules and the associated data flow are formalized as follows.

(1) ALA-based parameter adaptive optimization.

Let the original Brent crude oil price series be denoted as $x=\{x_{1},x_{2},\ldots ,x_{N}\}$. The first stage aims to address the optimal selection of the VMD hyperparameter pair (K,α).

The population search space of the Artificial Lemming Algorithm (ALA) is defined as $\Omega =\left[K_{\min},K_{\max}\right]\times \left[\alpha_{\min},\alpha_{\max}\right]$, where the state vector of the *i*-th individual is given by $x_{i}=\left(K_{i},\alpha_{i}\right)$, which directly corresponds to a candidate parameter configuration for VMD.

The objective function is defined as the minimum envelope entropy of the decomposition results. Accordingly, the ALA optimizer iteratively searches for the global optimum by solving the following minimization problem:

(9) $$\left(K^{*},\alpha^{*}\right)=\arg\min_{(K,\alpha )\in \Omega }\;\mathrm{EnEn}\left(\mathrm{VMD}(x;\;K,\alpha )\right),$$

where VMD(·) denotes the VMD operator and EnEn(·) denotes the envelope entropy.

In this process, ALA serves as the outer optimizer that dynamically updates the parameter candidates, while VMD acts as the inner evaluator that feeds back the decomposition quality.

(2) Signal adaptive decomposition.

Using the optimal parameter set $\left(K^{*},\alpha^{*}\right)$ obtained in Stage 1, the original non-stationary series x is decomposed via VMD. This operation maps the original signal space into an orthogonal subspace composed of $K^{*}$ intrinsic mode functions (IMFs):

(10) $$x\left(t\right)=\sum_{k=1}^{K^{*}}u_{k}\left(t\right).$$

The resulting subseries effectively separate high-frequency noise, medium-term fluctuations, and long-term trends embedded in the original data, thereby substantially reducing signal non-stationarity and structural complexity.

(3) Parallel CASA forecasting.

For each decomposed component $u_{k}$, an independent CASA-based forecasting channel is constructed. First, a sliding-window strategy is employed to reconstruct the one-dimensional time series into supervised learning samples. Specifically, for the *k*-th mode, the input feature matrix at time *t* is defined as

(11) $$X_{t}^{\left(k\right)}=\left[u_{k}\left(t-w+1\right),\;u_{k}\left(t-w+2\right),\;\ldots ,\;u_{k}\left(t\right)\right],$$

where *w* denotes the window length.

Subsequently, a nonlinear mapping function is constructed. The *k*-th CASA model extracts local morphological features from $X_{t}^{\left(k\right)}$ through its internal CNN autoencoder and outputs the one-step-ahead forecast:

(12) $${\hat{u}}_{k}\left(t+1\right)={\mathrm{CASA}}_{k}\left(X_{t}^{\left(k\right)}\right).$$

(4) Nonlinear ensemble aggregation and output.

Given that VMD satisfies the completeness constraint of signal reconstruction, i.e., $x\left(t\right)=\sum_{k=1}^{K^{*}}u_{k}\left(t\right)$, the final Brent crude oil price forecast can be obtained by linearly aggregating the predicted values of all subcomponents:

(13) $$\hat{x}\left(t+1\right)=\sum_{k=1}^{K^{*}}{\hat{u}}_{k}\left(t+1\right).$$

In summary, the proposed framework ensures optimal signal decomposition through ALA-driven adaptive parameter optimization, achieves effective stabilization of non-stationary price dynamics via VMD, and enhances sensitivity to localized structural patterns through CASA-based forecasting. The ALA-VMD-CASA framework integrates adaptive optimization, multi-scale signal decomposition, and attention-based prediction, and alleviates the situations of parameter sensitivity, cross-scale interaction, and noise interference. This integration enables the model to perform stable prediction, which is applied to analyze crude oil price series with strong fluctuation and non-stationary characteristics.

## 3. Experiments and Results

### 3.1. Data Source and Preprocessing

**Figure 3:**
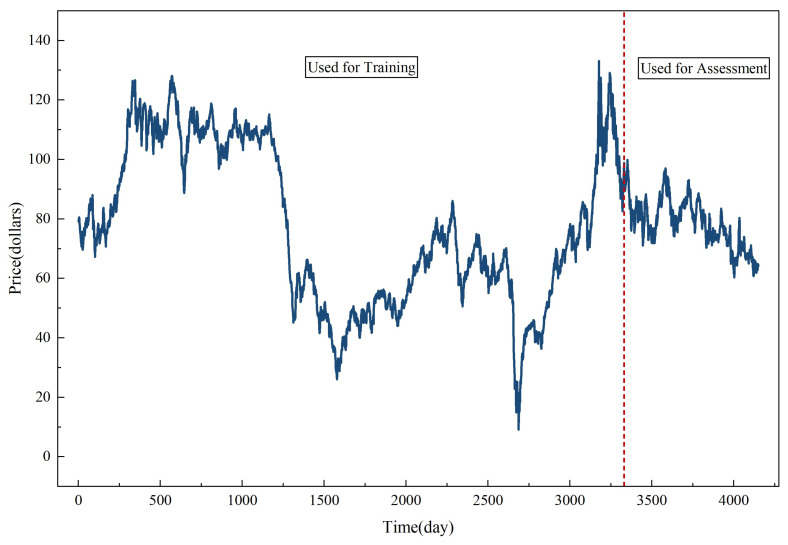
The daily spot price series of Brent crude oil from 2010 to 2025 and the data partition for training and testing. The vertical dashed line indicates the boundary between the training set and the testing set.

The data source for the empirical analysis is the Federal Reserve Economic Data (FRED) database. The Brent crude oil price series (DCOILBRENTEU) serves as a key global benchmark for international oil markets and is widely used in empirical energy economics and financial forecasting studies, ensuring both data reliability and economic relevance. The data sample consists of daily data from 4 January 2010 through 28 November 2025. After removing missing values, the dataset contains a total of 4150 valid daily records. Figure 3 presents the daily Brent crude oil price series over the sample period and illustrates the chronological partition between the training and testing sets.

**Figure 4:**
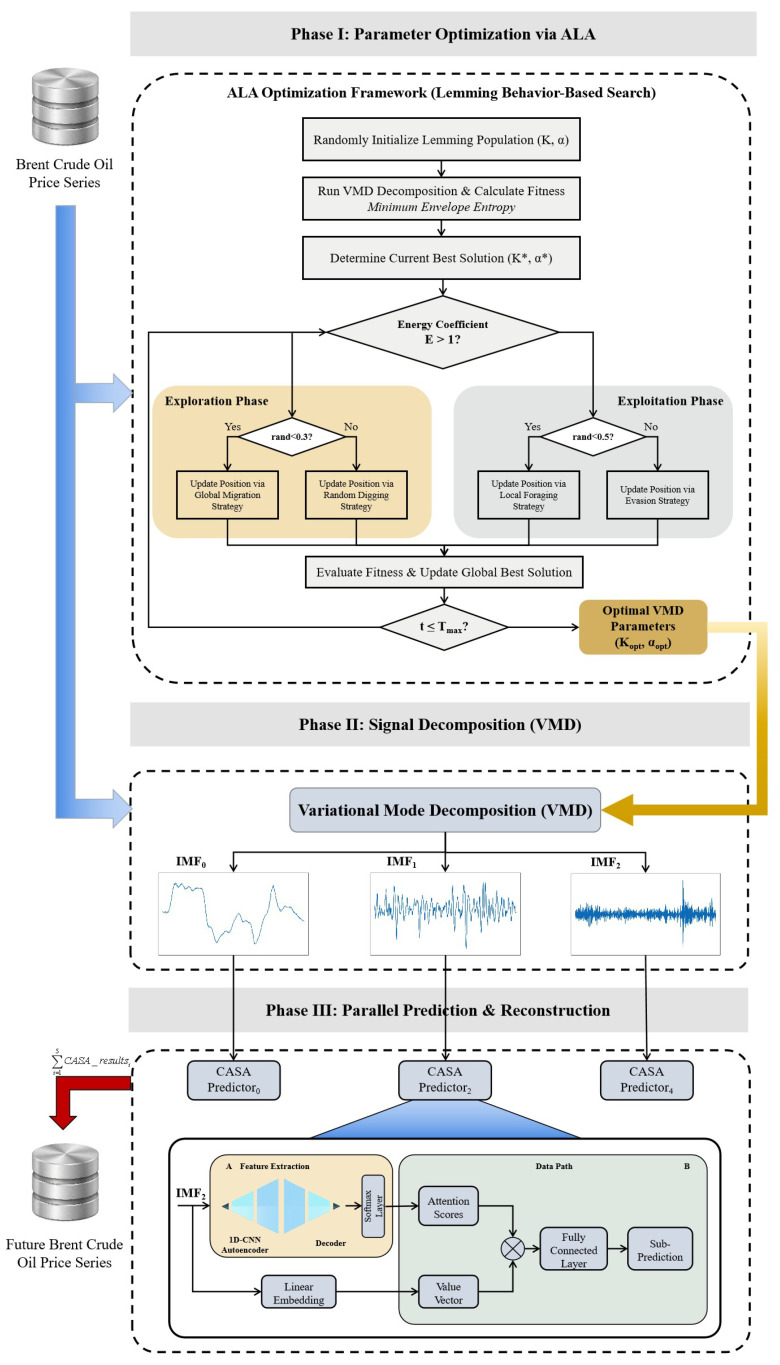
Overall architecture of the proposed ALA-VMD-CASA framework. The framework consists of three phases: (I) adaptive parameter optimization of VMD using the Artificial Lemming Algorithm (ALA), (II) multi-scale signal decomposition via VMD, and (III) parallel forecasting of decomposed components using CASA predictors followed by reconstruction.

Similar to time series prediction research, the dataset is divided into training set and test set according to time for out-of-sample performance evaluation [29]. The first 3330 observations (from 4 January 2010 to early October 2022, a total of approximately twelve years) are used for model estimation and parameter calibration. The remaining 820 observations (from 10 October 2022 to 28 November 2025, approximately three years) are used for out-of-sample testing. This rolling forward division makes the data have time series ordering, and there is no information leakage between training and evaluation. Such a setting can evaluate the model prediction ability in a prediction situation closer to the real world. The overall architecture of the proposed ALA-VMD-CASA forecasting framework is illustrated in Figure 4.

### 3.2. Benchmark Models and Evaluation Metrics

To evaluate the forecasting performance of the proposed ALA-VMD-CASA framework, we compare it with a set of benchmark models including ARIMA, the random-walk benchmark (RW), the random-walk-with-drift benchmark (RWWD), LSTM, GRU, VMD-GRU, Transformer, and Informer. The inclusion of RW and RWWD is essential because random-walk-type forecasts have become increasingly important benchmark references in the evaluation of financial and commodity price forecasting models [16,17]. In the daily Brent crude oil price setting considered here, RW corresponds to the canonical end-of-day no-change forecast, namely, the forecast for time t+1 is the observed price at time *t*. RWWD extends this benchmark by allowing for a constant drift term estimated from the training sample. Reporting both RW and RWWD enables us to compare the proposed framework not only against the canonical no-change benchmark for daily financial prices, but also against a closely related drift-adjusted naive benchmark. The selected benchmark set therefore covers traditional statistical methods, random-walk-type naive forecasts, standard deep learning architectures, decomposition-based hybrid methods, and recent attention-based forecasting models, providing a more comprehensive basis for evaluating the effectiveness and robustness of the proposed framework.

All benchmark models are implemented under the same experimental configuration to ensure comparability. The length of the sliding window is 12. The recurrent neural network uses 100 hidden units, the batch size is 64, and it is trained for 100 epochs with a learning rate of 0.0001. The signal decomposition based on VMD is implemented by vmdpy. Under comparable computational configurations, the Transformer and Informer models are trained using only the training set, and the reported evaluation results are calculated based on a separate test set, so that the comparison can reflect the real out-of-sample prediction performance. Among these controlled conditions, the difference in performance is mainly due to the difference in the modeling ability of each method, not the difference in the training procedure, which helps to make the experimental results reliable and reproducible. To prevent information leakage, all models strictly use only historical observations available up to time *t* when generating forecasts for time t+h; no contemporaneous or future information is included in the input features at any stage of the forecasting pipeline.

To quantitatively evaluate forecasting accuracy from multiple perspectives, four widely adopted statistical error metrics are employed: Mean Absolute Error (MAE), Root Mean Squared Error (RMSE), Mean Absolute Percentage Error (MAPE), and the coefficient of determination ($R^{2}$). These metrics are defined as follows:

(14) $$\mathrm{MAE}=\frac{1}{n}\sum_{i=1}^{n}\left|y_{i}-{\hat{y}}_{i}\right|,$$

(15) $$\mathrm{RMSE}=\sqrt{\frac{1}{n}\sum_{i=1}^{n}{\left(y_{i}-{\hat{y}}_{i}\right)}^{2}},$$

(16) $$\mathrm{MAPE}=\frac{1}{n}\sum_{i=1}^{n}\left|\frac{y_{i}-{\hat{y}}_{i}}{y_{i}}\right|\times 100\%,$$

(17) $$R^{2}=1-\frac{\sum_{i=1}^{n}{\left(y_{i}-{\hat{y}}_{i}\right)}^{2}}{\sum_{i=1}^{n}{\left(y_{i}-\bar{y}\right)}^{2}},$$

where $y_{i}$ denotes the observed value, ${\hat{y}}_{i}$ represents the predicted value, $\bar{y}$ is the mean of the observed values, and *n* is the number of samples in the testing set. Lower values of MAE and RMSE indicate smaller prediction errors, while an $R^{2}$ value closer to unity implies stronger explanatory power with respect to price variations.

### 3.3. Results and Discussion

The empirical results include four parts: convergence analysis of the optimization process, comparison of model prediction performance, inspection of the time evolution of prediction errors, and evaluation of statistical significance.

Before comparing performance, first study the convergence characteristics of VMD parameter optimization based on ALA.

**Figure 5:**
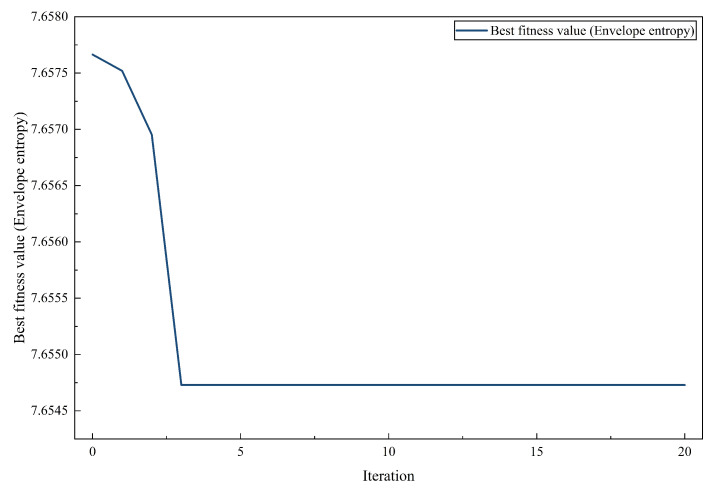
Convergence curve of the ALA-based VMD parameter optimization process (envelope entropy). The fitness value decreases rapidly in the early iterations and stabilizes thereafter, indicating fast convergence and stable search behavior of the ALA.

The optimal fitness of envelope entropy in Figure 5 changes during the optimization iteration. The curve drops sharply at first and then quickly stabilizes, which indicates that ALA can quickly reach a good solution and can also maintain a stable trajectory in the subsequent process.

In several iterations, the ALA algorithm is fast and stable, maintaining the balance between exploration and exploitation. This is very important for the number of discrete modes of VMD and the optimization of continuous penalty parameters. A smooth convergence curve means that there is no large oscillation or early stagnation in optimization, which often occurs when meta-heuristic algorithms search in heterogeneous parameter spaces. Since VMD decomposition is sensitive to the parameter set, stable convergence is necessary for consistent decomposition results after multiple runs.

#### 3.3.1. Comparative Performance Analysis

Table 1 presents the one-step-ahead prediction results of Brent crude oil prices by all candidate models. Four commonly used indicators are used to evaluate model performance: $R^{2}$, MSE, RMSE, MAE, and MAPE. It can be seen from the comparison results that there are differences among traditional statistical methods, traditional deep learning architectures, decomposition-based hybrid models and the proposed framework.

First, ARIMA(2,0,2), RW, and RWWD serve as benchmark models. Among them, RW corresponds to the canonical end-of-day no-change forecast for daily financial prices, while RWWD represents a closely related random-walk benchmark with drift [16,17]. The $R^{2}$ values of these three models are all about 0.967, and the MSE is about 2.24. This result indicates that, in daily crude oil price forecasting, the no-change or random-walk-type benchmark is difficult to surpass, which is consistent with the efficient market view and recent oil forecasting literature [16,17,24]. When directly applied to the original price series, standalone deep learning models are not always superior to the benchmark methods. The $R^{2}$ of LSTM and GRU are 0.942 and 0.950 respectively, and the MSE is 3.775 and 3.238, both underperforming RW. This shows that recurrent neural networks have difficulty capturing stable information from fluctuating and non-stationary prices. The attention mechanism narrows the gap. The $R^{2}$ obtained by Transformer and Informer are 0.961 and 0.959, which are close to those of the benchmark models, but their MSE values are still slightly higher. These results suggest that without appropriate signal preprocessing, even advanced deep learning models may fail to outperform the canonical random-walk/no-change benchmark [16,24]. At the same time, these models directly act on the original price sequence without clearly distinguishing multi-scale fluctuations from noise, which may constrain further performance gains. Importantly, these results show that none of the conventional statistical or deep learning benchmark models consistently outperform the random-walk/no-change benchmark under the present experimental design, highlighting the strength of this benchmark in daily crude oil price forecasting.

**Figure 6:**
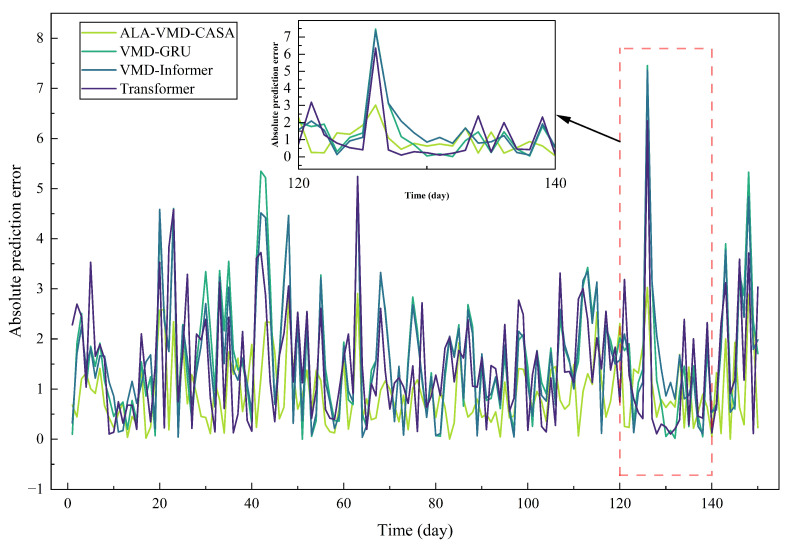
Absolute prediction errors of different forecasting models over the test period. The inset plot provides a zoomed-in view for t=120–140, where the proposed ALA-VMD-CASA model exhibits notably lower error magnitudes during abrupt price fluctuations.

Figure 6 shows the absolute prediction errors of all the comparison models during the test period to study the evolution of the errors. When the crude oil price fluctuates, the prediction errors of all methods change.

**Figure 7:**
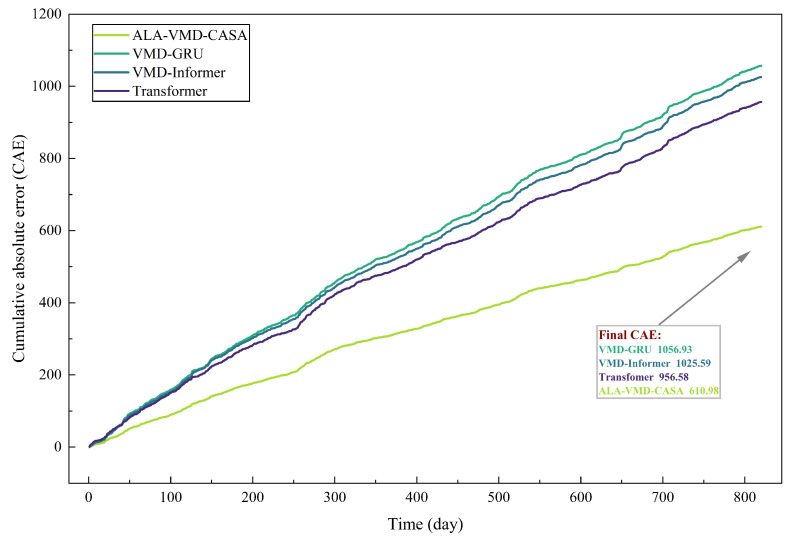
Cumulative absolute error (CAE) curves of different forecasting models over the test period. The proposed ALA-VMD-CASA model demonstrates the slowest growth of cumulative error, indicating superior long-term forecasting stability.

Pointwise error metrics can describe short-term accuracy, but they cannot fully reflect the long-term cumulative situation of prediction errors. To make up for this shortcoming, Figure 7 shows the CAE trajectories of all evaluation models in the test samples.

The comparison between Figure 6 and Figure 7 highlights the difference between short-term error control and long-term error accumulation. Short-term robustness reflects the model’s ability to handle sudden market fluctuations, while long-term reliability depends on whether small forecast errors accumulate over time. Thus, models that lack structural stability may exhibit considerable error accumulation when evaluated over longer periods of time.

To evaluate whether the observed performance differences are statistically significant differences caused by non-sample variance, a paired DM test is carried out between the ALA-VMD-CASA and the comparison models. The DM test tests the null hypothesis that the prediction accuracies are equal by testing whether the expected value of the loss difference sequence is not equal to zero. This framework is applicable to the comparison of time series prediction models and can handle the serial correlation and heteroscedasticity in prediction errors. In this study, the DM test is carried out under the condition of one-step-ahead prediction, using MAE and MSE as loss functions, and can be robustly evaluated under the standards of absolute error and squared error. Table 2 shows that the ALA-VMD-CASA is significantly better than all the benchmark models. In all pairwise comparisons, the DM statistic is negative, which indicates that the prediction error of the proposed framework is smaller. The relevant *p*-value is less than $1\times 10^{-16}$, so the null hypothesis of equal prediction accuracy is rejected at the 1% significance level. Due to a large number of pairwise comparisons, we test whether the statistical conclusion is sensitive to the multiple test effect. Holm’s stepwise method and Benjamini–Hochberg FDR correction are adopted. The adjusted conclusions of the two methods are unchanged, which means that the superior performance of ALA-VMD-CASA is not due to multiple comparisons, but the stable improvement of prediction accuracy.

Thirdly, the decomposition–prediction–reconstruction paradigm can separate multi-scale components to improve prediction accuracy, and its advantages vary due to different model combinations. In Table 1, VMD-GRU is better than GRU. The $R^{2}$ increases from 0.950 to 0.959, and MSE decreases from 3.238 to 2.785, showing that VMD reduces the complexity of modeling and also improves the prediction ability of decomposed subsequences. VMD-Informer is slightly better than Informer, and MSE is reduced from 2.676 to 2.632. And VMD-Transformer is not obviously better than Transformer: although the $R^{2}$ is almost the same (0.961 vs. 0.961), MSE has a slight increase, changing from 2.522 to 2.593. These results show that decomposition alone cannot improve the generalization ability; effectiveness is related to the decomposition mechanism and the prediction architecture. Finally, the ALA-VMD-CASA framework has the best overall performance, with an $R^{2}$ reaching 0.986, and the MSE, RMSE, MAE, and MAPE are reduced to 0.911, 0.954, 0.745, and 0.952, respectively. Compared with the best standalone model Transformer (MSE = 2.522), the proposed framework reduces MSE to 0.911, an improvement of about 63.9%. Compared with the similar VMD–Informer (MSE = 2.632), it further reduces MSE by about 65%. The income comes from VMD multi-scale decomposition, ALA adaptive parameter optimization, as well as CASA local structure pattern and key fluctuation dynamics. From the application perspective, the MAE drops to 0.745, which reduces the average deviation between the predicted price and the observed price, thus providing more reliable quantitative support for crude oil market price prediction, risk management, and trading strategy development.

**Figure 8:**
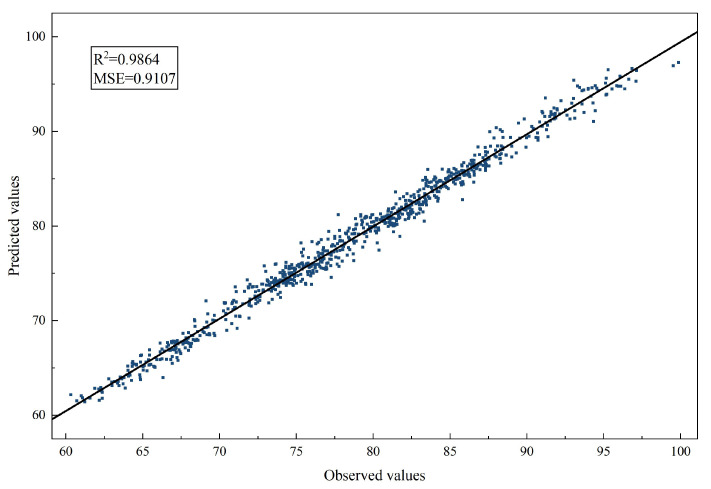
Scatter plot of predicted versus observed Brent crude oil prices for the ALA-VMD-CASA model. The tight clustering of data points around the fitted regression line indicates a high degree of consistency between predictions and observations.

To further assess the goodness of fit, Figure 8 depicts the relationship between predicted and observed Brent crude oil prices generated by the ALA-VMD-CASA model, along with the corresponding fitted regression line. The tight clustering of data points around the fitted line demonstrates a strong linear association and a high degree of consistency between model predictions and observations. This pattern indicates that the proposed model does not exhibit systematic overestimation or underestimation across different price levels.

**Figure 9:**
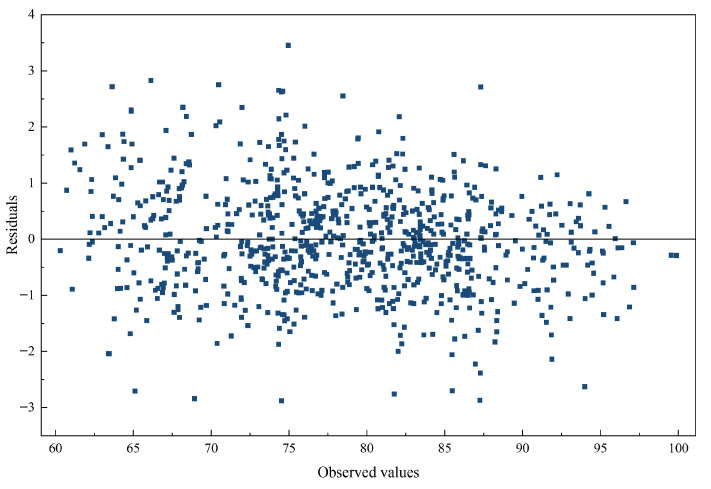
Residual distribution of the ALA-VMD-CASA model with respect to observed values. The residuals are approximately centered around zero with no discernible systematic pattern, suggesting that the model has effectively captured the major nonlinear dynamics in the data.

Figure 9 displays the associated residual distribution with respect to the observed values. The residuals are generally centered around zero and do not exhibit obvious systematic patterns, suggesting that prediction errors remain stable across the examined range. The lack of visible structure in the residual series also indicates that the principal nonlinear characteristics of the data have been effectively captured by the model. From the perspective of financial market applications, the substantial reduction in forecasting errors achieved by the ALA-VMD-CASA framework has practical implications. More accurate predictions lead to narrower confidence intervals for price forecasts, more reliable Value-at-Risk assessments, and clearer signals for algorithmic trading strategies. By reducing the uncertainty embedded in the residual series, the framework allows market participants to respond to market changes with greater precision, which is particularly valuable during periods of heightened volatility or structural shifts. This finding carries important implications for the broader literature. Specifically, it calls for caution regarding the common claim that machine learning models uniformly outperform traditional statistical benchmarks, particularly in studies where the random-walk or no-change benchmark is absent or improperly specified. By explicitly incorporating RW and RWWD as benchmarks and adopting a rigorous evaluation design, this study demonstrates that only the ALA-VMD-CASA framework achieves consistent and statistically significant outperformance over these competitive naive baselines.

#### 3.3.2. Ablation Study Analysis

Table 3 presents the results of ablation experiments under different model configurations, which are used to test the contributions of each part of the main framework separately.

The effectiveness of the parameter optimization strategy is first evaluated by comparing ALA-VMD-CASA with variants employing other meta-heuristic optimizers. Under the same decomposition and optimization conditions, the roles of the prediction modules are compared between ALA-VMD-DLinear and ALA-VMD-CASA. After replacing DLinear with CASA, the MSE changes from 2.404 to 0.911, a decrease of 62.1%. DLinear can capture long-term trends, but it is not good at fine price fluctuations. CASA combines convolutional feature extraction and attention mechanism, and is more effective in identifying structural patterns in crude oil price changes and volatility dynamics.

The importance of the decomposition stage is evaluated by comparing the CASA benchmark model with the complete proposed framework. Introducing adaptive decomposition can improve the prediction performance, which shows that the decomposition–prediction–reconstruction framework is effective in energy price prediction. Dividing the highly non-stationary crude oil price series into stable components reduces the complexity of the learning task and also improves the prediction accuracy and generalization ability.

The evidence of comprehensive comparison and ablation experiments indicates the key role of decomposition quality and prediction architecture design in crude oil price prediction. Combining adaptive optimized decomposition with attention-based prediction mechanism can capture multi-scale information, and the proposed hybrid framework has a significant improvement in accuracy and stability, becoming a practical tool for price prediction in the energy market.

**Figure 10:**
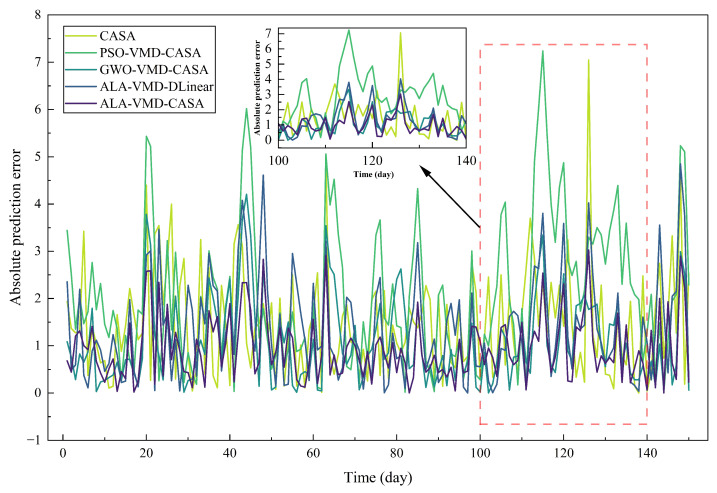
Absolute prediction errors of different ablation models over the test period. Models incorporating adaptive parameter optimization and signal decomposition exhibit more controlled error trajectories, while PSO-VMD-CASA displays substantial instability.

**Figure 11:**
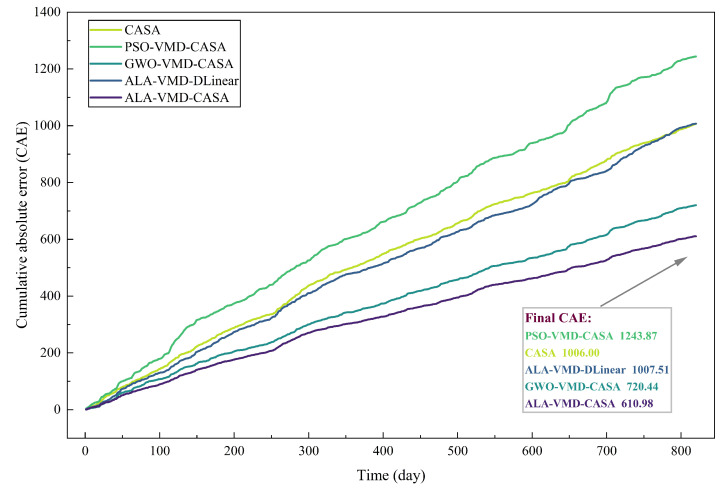
Cumulative absolute error (CAE) curves of different ablation models over the test period. ALA-VMD-CASA achieves the slowest cumulative error growth, confirming that its architectural components jointly contribute to long-term forecasting stability.

Although the preceding tables and figures characterize overall forecasting performance differences among competing models, we cannot completely distinguish the effects of adaptive optimization, signal decomposition, and attention mechanism, nor can we show how these components affect the evolution of prediction errors over time. To explore these situations, Figure 10 and Figure 11 respectively present the absolute prediction errors and cumulative absolute errors of each ablation variant. This additional analysis can more clearly reveal the internal mechanism behind the excellent performance of the ALA-VMD-CASA framework.

There are differences in the short-term error behavior of the ablation variants in Figure 10. The model with adaptive parameter optimization and signal decomposition has a relatively smooth error trajectory, especially when the price fluctuation increases. The model without these components has frequent sudden error peaks, which indicates that it is more sensitive to sudden market disturbances. The PSO-VMD-CASA variant is very unstable, and there are obvious prediction error fluctuations sometimes. This behavior indicates that an inappropriate or unstable parameter set may make the signal decomposition inconsistent, thereby amplifying the short-term prediction error.

The short-term deviation in Figure 11 accumulates over time. The CAE curve shows that the model without adaptive optimization or an effective attention mechanism accumulates prediction errors relatively fast. The local prediction is inaccurate and not well corrected, and it is passed on in subsequent predictions, making the overall error increase. This pattern reflects that when decomposition is stable or the feature weight mechanism is insufficient, there are limitations in the structure that controls error accumulation. The cumulative error growth is the lowest and the cumulative pattern is more stable when ALA-VMD-CASA is tested. This display framework makes the point-by-point prediction accuracy improved and the overall error generation dynamics reduced. By stabilizing signal decomposition and re-evaluating the variance in the subtraction parameter selection to highlight local feature information, the constructed framework can avoid the gradual accumulation of prediction errors. Therefore, this model has stronger long-term stability and robustness.

### 3.4. Robustness Analysis

The role of each component in the error dynamics in the pre-ablation experiment. But additional analysis is needed to verify whether the advantages still exist under different experimental conditions. Crude oil prices have obvious characteristics of non-stationarity, state transition and structural breaks. The conclusion obtained from the single training–test split may be affected by sample-specific factors.

To solve the problem, three ways are adopted to test robustness: time robustness is evaluated by rolling with an extended window, as well as performance on multiple predicted horizons, and sensitivity to random initialization. These tests are used to see whether the ALA-VMD-CASA framework can maintain stable and reliable prediction performance under different market conditions, predicted horizons, and random training conditions.

#### 3.4.1. Expanding-Window Walk-Forward Robustness Test

An expanding-window walk-forward evaluation procedure is adopted. The process begins with an initial training sample of length $N_{0}$ constructed from the earliest observations. The model is then used to generate forecasts for the following *h*-day horizon. Once the actual observations become available, they are appended to the training set, and the estimation window is moved forward by a step size *s*. This procedure continues iteratively until the entire testing period has been evaluated.

Extending the forward evaluation of window scrolling is to check the time robustness under market conditions. Different from the fixed partition of training and test sets, this method relies on gradually expanding the training samples by adding new observations. So this evaluation method is more in line with the actual prediction scenario, and the model is updated regularly with new information. The result in Table 4 is that ALA-VMD-CASA has the lowest average prediction error in the rolling window, and the standard deviation of the error index is much smaller than that of the benchmark model. This reduced variability enables the proposed framework to stably make predictions at different times, not just perform well in specific market periods.

With the expansion of training samples, the performance advantage of ALA-VMD-CASA is relatively obvious, which means that it can absorb new information without obvious performance degradation or overfitting. In order to verify whether this advantage is the same in different evaluation windows, Table 5 lists the win rate statistics based on MSE. ALA-VMD-CASA has a win rate of 1 in all rolling windows, while no benchmark model has the minimum MSE in a single window.

#### 3.4.2. Multi-Horizon Forecasting Robustness

Evaluating models across different horizons can test robustness because as the horizon becomes longer, uncertainty and errors will increase. Table 6 shows that the errors of all models increase from 1 step to 10 steps, which reflects the uncertainty of oil price dynamics. The error of ALA-VMD-CASA is still the lowest at each horizon. The advantage of ALA-VMD-CASA becomes stronger as the horizon extends, indicating that it can resist cumulative errors and perform well in short, medium, and long terms.

The strong multi-step forecasting performance of this framework is related to its decomposition–forecasting–reconstruction design. Before forecasting, the original time series is decomposed into different components to reduce the mutual influence between different time scales and avoid the impact of short-term errors on long-term forecasting. Therefore, the model can maintain structural consistency under different forecasting horizons, thus providing more reliable medium- and long-term forecasts in a volatile market.

**Figure 12:**
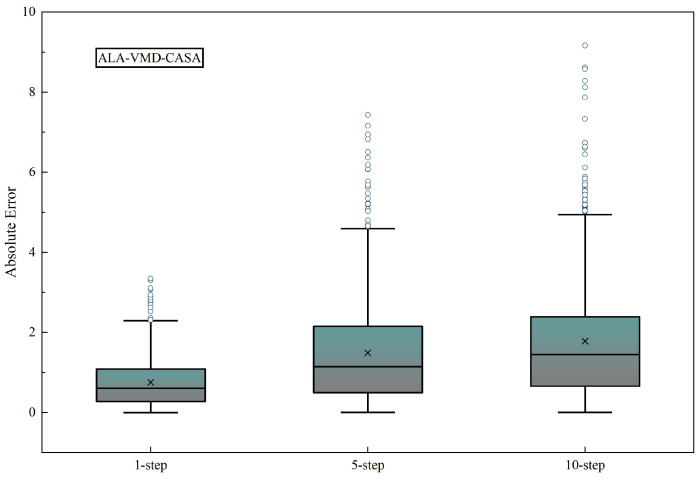
Boxplots of absolute forecasting errors under different prediction horizons for the ALA-VMD-CASA model. As the forecasting horizon extends, both the median error and dispersion increase progressively, while the distributions remain relatively concentrated.

To further examine the distributional characteristics of prediction errors across forecasting horizons, Figure 12 presents boxplots of absolute errors for the 1-step, 5-step, and 10-step forecasts. Even so, the error distributions remain relatively compact, indicating that the proposed framework maintains stable predictive behavior even when forecasts extend further into the future.

#### 3.4.3. Robustness to Random Initialization

Given that deep learning training processes and meta-heuristic optimization procedures may introduce stochastic variability, robustness with respect to random initialization is further examined. Under a fixed data partition, multiple independent experiments are conducted using different random seeds, and the mean and standard deviation of each error metric are reported. The corresponding results are presented in Table 7.

In Table 7, the performance differences between different seeds of ALA-VMD-CASA are relatively small. For example, the standard deviations of $R^{2}$, RMSE, and MAE are 0.000, 0.006, and 0.005, respectively, which indicates that the prediction performance is highly consistent. ALA-VMD-CASA has an advantage in the average error on the benchmark model. This shows that the improvement in performance is due to the stable model design and reproducibility, rather than relying on random initialization to take a chance.

## 4. Conclusions

This study explores the challenges in predicting Brent crude oil prices, including large uncertainties, multi-scale fluctuations, nonlinear dynamics, and irregular market fluctuations. The difficulties in overcoming methods to solve these problems lie in the sensitivity of signal decomposition parameters, the complex dynamics of multiple time scales, and the computational burden of long-sequence models. To address this, this study constructs a hybrid prediction framework, integrating ALA, VMD, and a prediction model based on CASA.

Empirical analysis of Brent crude oil spot price data shows that none of the benchmark models—including ARIMA, RW, RWWD, and various deep learning architectures—consistently outperform the random-walk/no-change benchmark. Only the ALA-VMD-CASA framework surpasses RW and RWWD, which strengthens the contribution of this study by demonstrating that the proposed model achieves genuine predictive advantages over both standard statistical and machine learning models and the appropriate random-walk-type naive benchmarks. The $R^{2}$ of this model is 0.986, and the MSE is 0.911, which is about 63.9% higher than the best single-model benchmark. The results of the ablation experiments show that the main components of the framework are complementary. ALA optimization makes the parameter selection in the decomposition stage tend to be stable. VMD splits the original sequence into multiple components and captures dynamic changes at different scales. The CASA module uses attention and linear components to enhance the model’s ability to identify local structural patterns.

Additional robustness tests confirm that the framework is reliable. There are cases of forward validation using rolling windows, using perfect winning rates, conducting multi-step forecasts (including 1-step, 5-step, 10-step), and repeating experiments with different random seeds. In various tests under different market conditions and prediction horizons, the model has always maintained stable and excellent prediction performance.

In the energy economy, more accurate forecasting and more stable error performance can make the model more effective in pricing in the changing crude oil market. Reliable forecasting can assist in carrying out risk management, investment decision-making, policy analysis and such affairs. Although the research results are quite exciting, there are still many directions for future research to explore. Perhaps what can be expanded is to apply this framework to high-frequency data, incorporate macroeconomic and geopolitical factors to make the economic explanation more powerful, and develop more computationally efficient optimization methods to promote large-scale pricing applications.

## Figures and Tables

**Table 1:** One-step-ahead forecasting performance comparison of different models on the Brent crude oil dataset.

Model	$R^{2}$	MSE	RMSE	MAE	MAPE
ARIMA(2,0,2)	0.967	2.238	1.496	1.137	1.454
RW	0.967	2.244	1.498	1.138	1.455
RWWD	0.967	2.244	1.498	1.138	1.455
LSTM	0.942	3.775	1.943	1.492	1.924
GRU	0.950	3.238	1.800	1.370	1.764
Transformer	0.961	2.522	1.588	1.212	1.567
Informer	0.959	2.676	1.636	1.284	1.643
VMD-GRU	0.959	2.785	1.669	1.289	1.648
VMD-Transformer	0.961	2.593	1.610	1.244	1.590
VMD-Informer	0.961	2.632	1.622	1.251	1.599
ALA-VMD-CASA	0.986	0.911	0.954	0.745	0.952

Note: This table reports the one-step-ahead out-of-sample forecasting results. Lower values of MSE, RMSE, MAE, and MAPE indicate better forecasting performance, while higher $R^{2}$ indicates better fit.

**Table 2:** Diebold–Mariano test results for pairwise forecast comparison.

Reference Model	Competing Model	Loss	DM Statistic	*p*-Value
ALA-VMD-CASA	ARIMA	MAE	−12.73	<$1\;\times \;10^{-16}$
ALA-VMD-CASA	RW	MAE	−12.82	<$1\;\times \;10^{-16}$
ALA-VMD-CASA	RWWD	MAE	−12.81	<$1\;\times \;10^{-16}$
ALA-VMD-CASA	GRU	MAE	−17.79	<$1\;\times \;10^{-16}$
ALA-VMD-CASA	Informer	MAE	−13.56	<$1\;\times \;10^{-16}$
ALA-VMD-CASA	LSTM	MAE	−20.01	<$1\;\times \;10^{-16}$
ALA-VMD-CASA	Transformer	MAE	−12.83	<$1\;\times \;10^{-16}$
ALA-VMD-CASA	VMD-GRU	MAE	−17.76	<$1\;\times \;10^{-16}$
ALA-VMD-CASA	VMD-Informer	MAE	−17.11	<$1\;\times \;10^{-16}$
ALA-VMD-CASA	VMD-Transformer	MAE	−17.05	<$1\;\times \;10^{-16}$
ALA-VMD-CASA	ARIMA	MSE	−11.09	<$1\;\times \;10^{-16}$
ALA-VMD-CASA	RW	MSE	−11.11	<$1\;\times \;10^{-16}$
ALA-VMD-CASA	RWWD	MSE	−11.11	<$1\;\times \;10^{-16}$
ALA-VMD-CASA	GRU	MSE	−12.60	<$1\;\times \;10^{-16}$
ALA-VMD-CASA	Informer	MSE	−11.59	<$1\;\times \;10^{-16}$
ALA-VMD-CASA	LSTM	MSE	−13.32	<$1\;\times \;10^{-16}$
ALA-VMD-CASA	Transformer	MSE	−11.33	<$1\;\times \;10^{-16}$
ALA-VMD-CASA	VMD-GRU	MSE	−12.41	<$1\;\times \;10^{-16}$
ALA-VMD-CASA	VMD-Informer	MSE	−12.33	<$1\;\times \;10^{-16}$
ALA-VMD-CASA	VMD-Transformer	MSE	−12.31	<$1\;\times \;10^{-16}$

Note: Negative DM statistics indicate that ALA-VMD-CASA achieves systematically lower forecast errors than the competing model. All *p*-values are below $1\times 10^{-16}$.

**Table 3:** Ablation study results for validating the contributions of the adaptive optimization, decomposition, and attention modules.

Model	$R^{2}$	MSE	RMSE	MAE	MAPE
CASA (Baseline)	0.963	2.515	1.586	1.226	1.570
GWO-VMD-CASA	0.980	1.320	1.149	0.879	1.128
PSO-VMD-CASA	0.944	3.790	1.947	1.517	1.940
ALA-VMD-DLinear	0.964	2.404	1.551	1.229	1.586
ALA-VMD-CASA	0.986	0.911	0.954	0.745	0.952

Note: CASA denotes the baseline model without signal decomposition or parameter optimization. GWO and PSO denote alternative meta-heuristic optimizers, and DLinear denotes an alternative forecasting module.

**Table 4:** Statistical results of the robustness test using the expanding-window walk-forward evaluation strategy.

Model (Mean ± Std)	$R^{2}$	MSE	MAE	MAPE (%)
Transformer	0.773±0.163	4.335±5.858	1.460±0.756	2.279±2.104
VMD-GRU	0.321±0.561	15.167±23.102	2.695±1.764	4.367±5.431
VMD-Informer	0.668±0.215	8.002±14.213	1.909±1.270	3.227±4.798
ALA-VMD-CASA	0.938±0.042	1.388±1.596	0.832±0.407	1.241±0.775

Note: Each metric is reported as mean ± standard deviation across multiple expanding-window forecasting periods.

**Table 5:** Win rate based on minimum MSE.

Model	Win Rate (by min MSE)
ALA-VMD-CASA	1
Transformer	0
VMD-GRU	0
VMD-Informer	0

Note: The win rate indicates the proportion of evaluation windows in which a model achieves the lowest MSE among all competing models.

**Table 6:** Forecasting performance comparison under different prediction horizons.

Horizon	Model	$R^{2}$	MSE	RMSE	MAE	MAPE (%)
1	ARIMA(2,0,2)	0.967	2.238	1.496	1.137	1.454
	RW	0.967	2.244	1.498	1.138	1.455
	RWWD	0.967	2.244	1.498	1.138	1.455
	Transformer	0.961	2.522	1.588	1.212	1.567
	VMD-GRU	0.959	2.785	1.669	1.289	1.648
	VMD-Informer	0.961	2.632	1.622	1.251	1.599
	ALA-VMD-CASA	0.986	0.911	0.954	0.745	0.952
5	ARIMA(2,0,2)	0.824	11.561	3.400	2.647	3.399
	RW	0.823	11.648	3.412	2.658	3.413
	RWWD	0.823	11.660	3.414	2.660	3.415
	Transformer	0.811	12.443	3.527	2.777	3.562
	VMD-GRU	0.904	6.292	2.508	1.926	2.487
	VMD-Informer	0.931	4.560	2.135	1.674	2.160
	ALA-VMD-CASA	0.943	3.884	1.971	1.495	1.912
10	ARIMA(2,0,2)	0.692	20.097	4.482	3.477	4.446
	RW	0.688	20.385	4.515	3.501	4.476
	RWWD	0.688	20.418	4.518	3.504	4.479
	Transformer	0.664	21.892	4.679	3.619	4.621
	VMD-GRU	0.850	9.795	3.130	2.427	3.126
	VMD-Informer	0.875	8.164	2.857	2.223	2.877
	ALA-VMD-CASA	0.922	5.332	2.309	1.767	2.248

Note: This table compares forecasting performance under 1-step, 5-step, and 10-step ahead prediction horizons using a direct forecasting strategy.

**Table 7:** Robustness analysis of the proposed framework under different random seeds.

Metric	ALA-VMD-CASA	Transformer	VMD-GRU	VMD-Informer
$R^{2}$	0.986±0.000	0.960±0.003	0.958±0.002	0.974±0.002
MSE	0.938±0.012	2.646±0.115	2.785±0.082	1.737±0.119
RMSE	0.969±0.006	1.627±0.037	1.669±0.026	1.319±0.041
MAE	0.753±0.005	1.279±0.043	1.282±0.025	1.042±0.038
MAPE (%)	0.959±0.007	1.640±0.045	1.641±0.026	1.337±0.070

Note: Each metric is reported as mean ± standard deviation across multiple independent runs with different random seeds.

## Data Availability

The datasets and codes generated or analyzed during the current study are available in the GitHub repository, https://github.com/Color-HE/ALA-VMD-CASA (accessed on 27 March 2026).
